# Prognosis and predictive factors of conversion surgery for initially unresectable advanced colorectal cancer

**DOI:** 10.1007/s00423-024-03374-0

**Published:** 2024-06-11

**Authors:** Gaku Ohira, Satoshi Endo, Shunsuke Imanishi, Toru Tochigi, Tetsuro Maruyama, Koichi Hayano, Michihiro Maruyama, Hisahiro Matsubara

**Affiliations:** Department of Frontier Surgery, Chiba university graduate school of medicine, 1-8-1 Inohana, Chuuou-ku, Chiba, 260-8670 Japan

**Keywords:** Colorectal cancer, Conversion surgery, Conversion rate, Neoadjuvant chemotherapy, Extrahepatic metastases

## Abstract

**Purpose:**

The aim of this study was to report the outcomes of conversion surgery for initially unresectable advanced colorectal cancer and to identify factors that enable successful conversion surgery.

**Methods:**

We compared the outcomes of patients with colorectal cancer with distant metastases, including extrahepatic metastases, who underwent upfront surgery, neoadjuvant chemotherapy, conversion surgery, and chemotherapy only at our department from 2007 to 2020. In addition, factors influencing the achievement of conversion surgery in patients who were initially unresectable were examined in univariate and multivariate analyses.

**Results:**

Of 342 colorectal cancer patients with distant metastases treated during the study period, 239 were judged to be initially unresectable, and 17 (conversion rate: 7.1%) underwent conversion surgery. The prognosis for the conversion surgery group was better than that of the chemotherapy only group but worse than that of the upfront surgery group. In the conversion surgery group, the recurrence-free survival after resection was significantly shorter than that upfront surgery group and neoadjuvant chemotherapy group, and no patients have been cured. Among patients who were initially unresectable, left-sided primary cancer and normal CA19-9 level were identified as independent factors contributing to the achievement of conversion surgery in a multivariate analysis.

**Conclusions:**

Although relapse after conversion surgery is common, and no patients have been cured thus far, overall survival was better in comparison to patients who received chemotherapy only. Among unresectable cases, patients with left-sided primary cancer and normal CA19-9 levels are likely to be candidates for conversion surgery.

**Supplementary Information:**

The online version contains supplementary material available at 10.1007/s00423-024-03374-0.

## Introduction

In cases of colorectal cancer, favorable outcomes can be anticipated through surgical intervention, even in the presence of distant metastases. The five-year survival rates after resection of liver, lung, and peritoneal metastases range from 45 to 60% [1], 30–68% [2], and 27–32% [3, 4], respectively. For unresectable cases, systemic chemotherapy is used, and with the remarkable advancements in chemotherapy, the median survival time is now almost three years [5]. Successful chemotherapy can render previously unresectable tumors resectable. Conversion surgery refers to surgical intervention that renders initially unresectable tumors resectable following treatment [6]. Given the potential for conversion surgery to improve prognoses [7], it is imperative to perform resection before the optimal window of opportunity elapses, even if the tumor is initially deemed unresectable. On the other hand, neoadjuvant chemotherapy is employed to improve the outcome after resection in resectable patients [8]. Because neoadjuvant chemotherapy and conversion surgery are similar in that surgery is performed after chemotherapy, they are often reported in a confusing manner. In this study, we report the results of upfront surgery, neoadjuvant chemotherapy, and conversion surgery for advanced colorectal cancer with distant metastasis, including extrahepatic metastasis, at our institution, and we aim to identify the factors that enable successful conversion surgery. We also raise questions regarding the definitions of conversion surgery and conversion rate.

## Methods

### Patients

Patients diagnosed with colorectal cancer who had distant metastasis and were treated at our department between 2007 and 2020 were included in this study. Resectability was determined based on two criteria: technical feasibility and preservation of vital organ function. The decision to proceed with resection was made after a group conference discussion. For cases deemed resectable, neoadjuvant chemotherapy was prescribed for patients with multiple organ or extensive distant lymph node metastases, as well as for cases with the possibility of exposing tumor tissue on the dissected surface (marginally resectable cases). The final decision was made by the attending physician, considering the patient’s overall health and preferences. Specific surgical procedures were determined by the hepatobiliary surgeon for liver metastases, by the respiratory surgeon for lung metastases, and the colorectal surgeon for the other cases. For peritoneal metastasis, the surgeon aimed for complete gross resection of the tumor, including resection of the dissemination nodule and concomitant resection of the surrounding organs if invasion was observed. Peritonectomy was not performed.

Patients were classified into four groups: those who underwent upfront surgery (Group S), those who received neoadjuvant chemotherapy before surgery (Group N), those who underwent conversion surgery (Group C), and those who received chemotherapy only because they were unresectable (Group I). The patients’ characteristics and prognoses were compared across these four groups.

### Study items

Data regarding age, sex, primary site, metastatic organ, number of metastases, tumor markers, treatment received (e.g., surgery, neoadjuvant chemotherapy, conversion surgery, etc.), overall survival after the start of treatment, and postresection recurrence-free survival for patients who underwent R0 or R1 resection were extracted from medical records. Then, univariate and multivariate analyses were conducted to investigate the features of cases that were amenable to conversion surgery among those that were initially deemed unresectable. The surgical procedures, postoperative mortality, and morbidity were also assessed in Groups S, N, and C. Postoperative complications were evaluated using the Clavien-Dindo classification (CD), and the prognosis and the occurrence of CD grade ≥ 3 complications were compared.

### Statistical analyses

We estimated survival curves using the Kaplan‒Meier method and compared them using a stratified log-rank test. For categorical variables, we used chi-squared and Fisher’s exact tests to compare clinical outcomes and proportions between groups as appropriate. For continuous variables, we used Wilcoxon tests. A logistic regression analysis was used for the multivariate analysis. *P* values of < 0.05 were considered to indicate statistical significance. Data were statistically analyzed using the JMP software program (version 16.2, SAS Institute Inc., Cary, NC, USA).

## Results

During the review period, 342 cases of colorectal cancer with distant metastasis were treated at our department, with 64 cases in Group S, 39 cases in Group N, and 239 cases initially deemed unresectable. Of the unresectable cases, 17 were in Group C, achieving a conversion rate of 7.1%, while 222 cases were in Group I. The background of each group is provided in Table 1. In Group C, all but one had left-sided primary cancer. Five patients had metastasis to multiple organs and underwent extended surgery. There were significantly more cases in Group C with R1 than in Groups S and N (*P* < 0.0001).

**Table 1:** Characteristics of CRC patients with distant metastases by group

	Group S^†^	Group *N*^‡^	Group C^§^	Group I^¶^
Gender				
Male	31 (48)	22 (56)	13 (76)	113 (51)
Female	33 (52)	17 (44)	4 (24)	109 (49)
Age (median)	65.5 (29–87)	64 (28–79)	63 (43–78)	67 (26–95)
Side of primary tumor				
Right side	20 (31)	5 (13)	1 (6)	84 (38)
Left side	44 (69)	34 (87)	16 (94)	138 (62)
Histological type				
Well or moderately differentiated adenocarcinoma	59 (92)	35 (90)	16 (94)	173 (79)
Others	5 (8)	4 (10)	1 (6)	47 (21)
Clinical T				
T1/T2/T3	23 (36)	15 (38)	6 (35)	57 (26)
T4a/T4b	41 (64)	24 (62)	11 (65)	164 (74)
Clinical N				
N-	18 (28)	5 (13)	3 (18)	35 (17)
N+	46 (72)	34 (87)	14 (82)	170 (83)
Metastatic organs				
1	57 (89)	34 (87)	10 (59)	75 (34)
2	5 (8)	5 (13)	7 (41)	96 (43)
More than 3	2 (3)	0 (0)	0 (0)	50 (23)
Hepatic metastasis				
Yes	44 (69)	19 (49)	13 (76)	150 (68)
No	20 (31)	20 (51)	4 (24)	72 (32)
Pulmonary metastasis				
Yes	4 (6)	6 (15)	3 (18)	95 (43)
No	6 (94)	33 (85)	14 (82)	127 (57)
Peritoneal metastasis				
Yes	15 (23)	4 (10)	2 (12)	83 (37)
No	49 (77)	35 (90)	15 (88)	129 (63)
Distant lymph node metastasis				
Yes	4 (6)	12 (31)	6 (35)	72 (33)
No	60 (94)	27 (69)	11 (65)	148 (67)
CEA				
Normal	24 (38)	11 (28)	6 (38)	38 (18)
Elevated	40 (62)	28 (72)	10 (62)	177 (82)
CA19-9				
Normal	37 (59)	22 (56)	10 (63)	71 (33)
Elevated	26 (41)	17 (44)	6 (37)	145 (67)
RAS status				
Wild	34 (71)	19 (53)	10 (67)	107 (57)
Mutant	14 (29)	17 (47)	5 (33)	79 (42)
Not evaluated	0 (0)	0 (0)	0 (0)	1 (1)
BRAF status				
Wild	41 (91)	33 (97)	13 (100)	241 (91)
Mutant	4 (9)	1 (3)	0 (0)	22 (8)
Not evaluated	0 (0)	0 (0)	0 (0)	1 (1)
Curability				
R0	62(97)	37(95)	10(59)	-
R1	2(3)	2(5)	7(41)	-

Data presented as n (%) or median

^†^Group S: Patients who underwent up-front surgery. ^‡^Group N: Patients who received neo-adjuvant chemotherapy before surgery. ^§^ Group C: Patients who underwent conversion surgery

^¶^Group I: Patients who received chemotherapy only

The surgical procedures of Groups S, N, and C are listed in Table 2. With regard to the surgical procedure for liver metastases, extended hepatic resection with ≥ 4 segments was performed more frequently in Group C. The postoperative mortality and morbidity are shown in Table 3. There was 1 patient each in Groups S and C who died of complications, but there was no significant difference between the groups. The complication rates were also not significantly different between the groups.

**Table 2:** Surgical procedure for up-front surgery group, neoadjuvant chemotherapy group, and conversion group

	Group S^†^	Group *N*^‡^	Group C^§^	*P* value
Hepatectomy				0.0033
none	20(31)	21(54)	4(24)	
Less than 3 segmentectomy	40(63)	15(38)	7(41)	
4 or more segmentectomy	4(6)	3(8)	6(35)	
Lung resection				0.2023
none	61(95)	33(85)	15(88)	
Less than lobectomy	2(3)	5(13)	1(6)	
More than lobectomy	1(2)	1(3)	1(6)	
Peritoneal resection				0.1677
None	50(78)	35(90)	15(88)	
Nodule resection	11(17)	1(3)	1(6)	
Nodule resection with invaded organ(s)	3(5)	3(8)	1(6)	
Resection of other organ(s)				
none	54(84)	21(54)	9(53)	
yes	10(16)	18(46)	8(47)	

Data presented as n (%)

^†^Group S: Patients who underwent up-front surgery. ^‡^Group N: Patients who received neo-adjuvant chemotherapy before surgery. ^§^ Group C: Patients who underwent conversion surgery

**Table 3:** Mortality and morbidity after R0/R1 resection in up-front surgery group, neoadjuvant chemotherapy group, and conversion group

	Group S^†^	Group *N*^‡^	Group C^§^	*P* value
Mortality				0.3681
Yes	1(2)	0	1(6)	
No	63	39	16	
Morbidity (Clavien-Dindo classification ≧ 3)				0.4452
Yes	17(27)	15(38)	6(35)	
No	47(73)	24(62)	11(65))	

Data presented as n (%)

^†^Group S: Patients who underwent up-front surgery. ^‡^Group N: Patients who received neo-adjuvant chemotherapy before surgery. ^§^ Group C: Patients who underwent conversion surgery

**Fig. 1:**
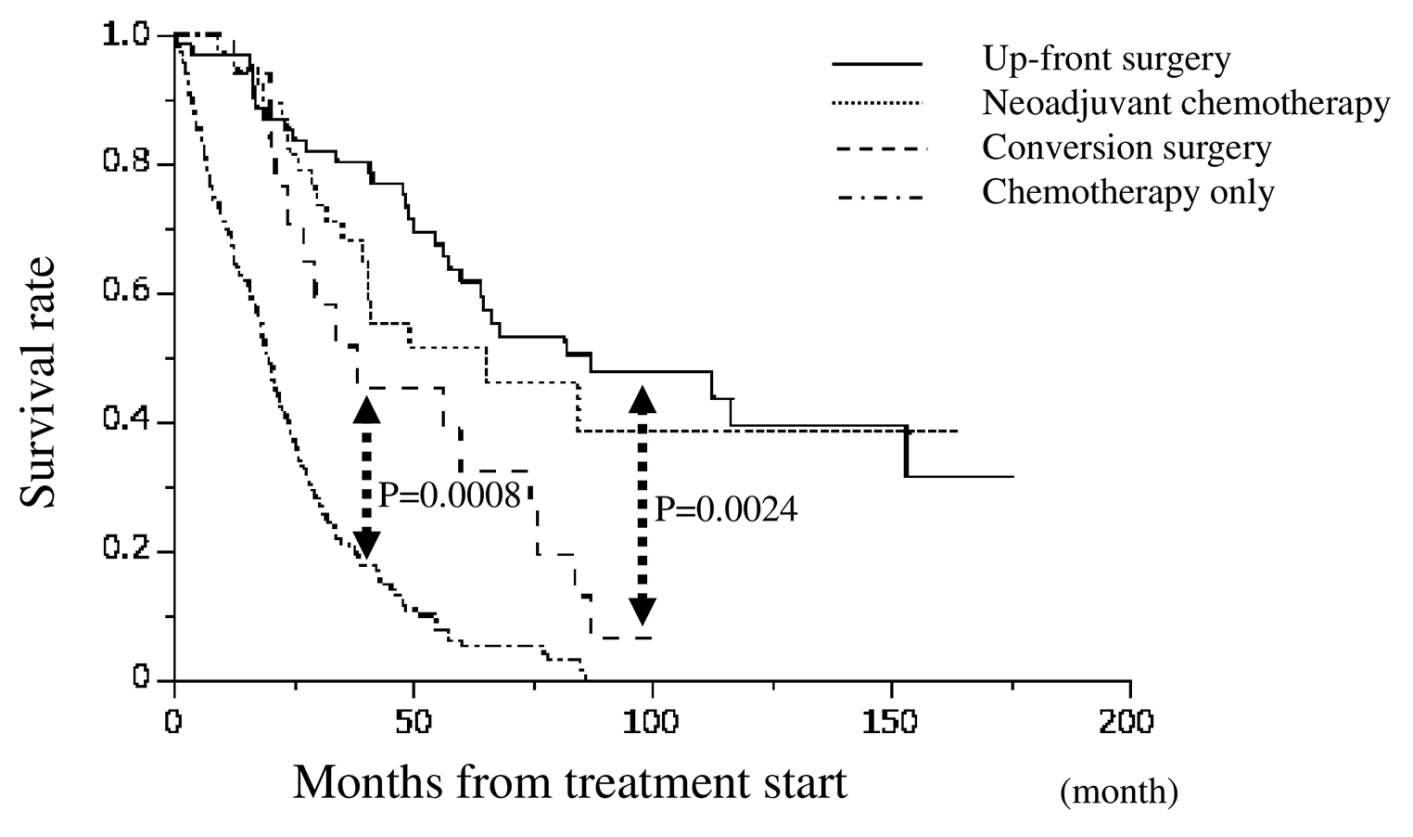
Survival curves from start of treatment for each group. Up-front surgery group had a significantly better prognosis than conversion surgery group (*P* = 0.0024, log rank test), while conversion surgery group had a significantly better prognosis than chemotherapy only group (*P* = 0.0008, lor rank test)

**Fig. 2:**
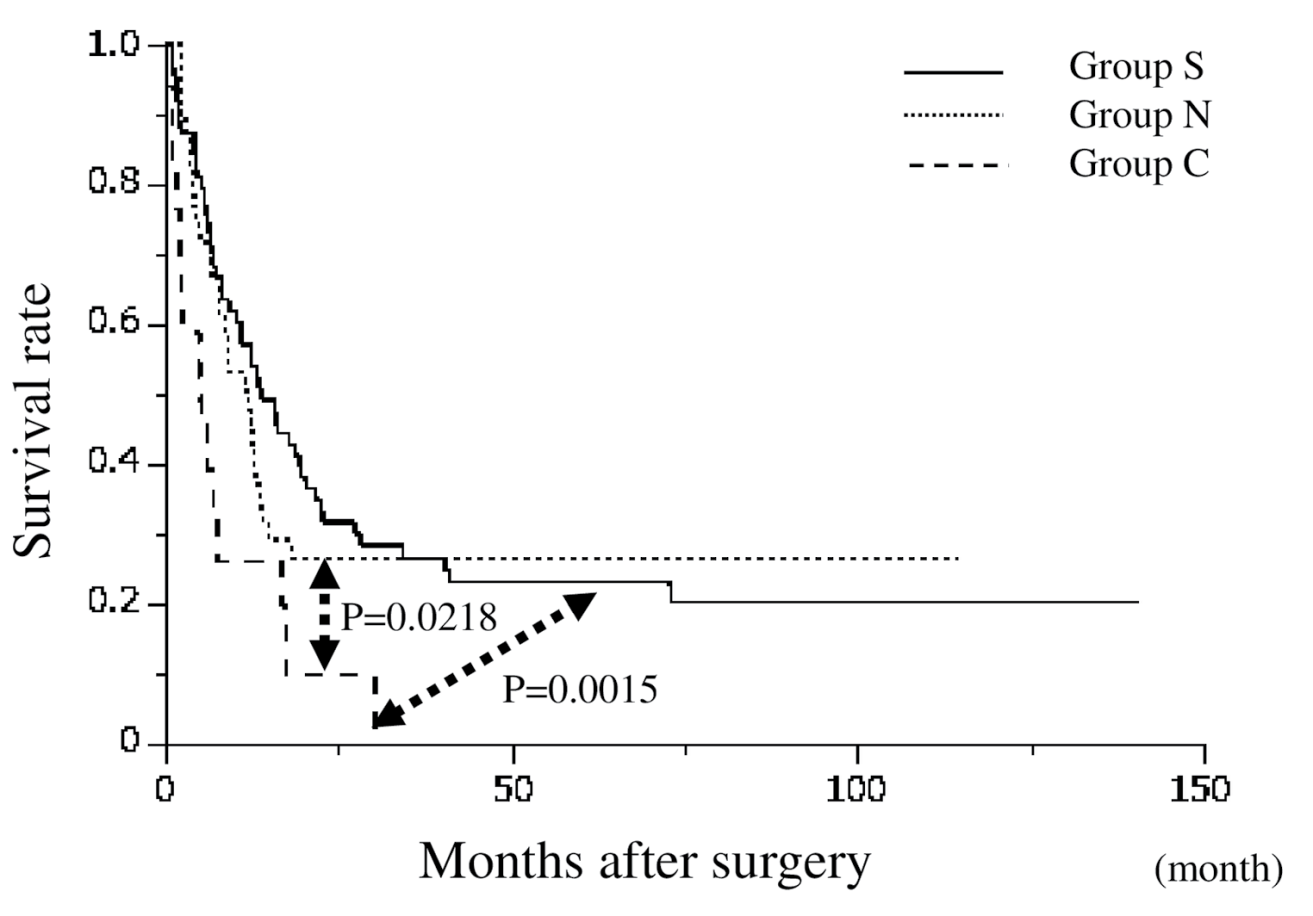
Relapse-free survival curves from R0 or R1 resection for each group. Conversion group had a significantly shorter RFS than Up-front surgery group and Neoadjuvant chemotherapy group (*P* = 0.0015 and 0.0218, respectively, log rank test)

**Fig. 3:**
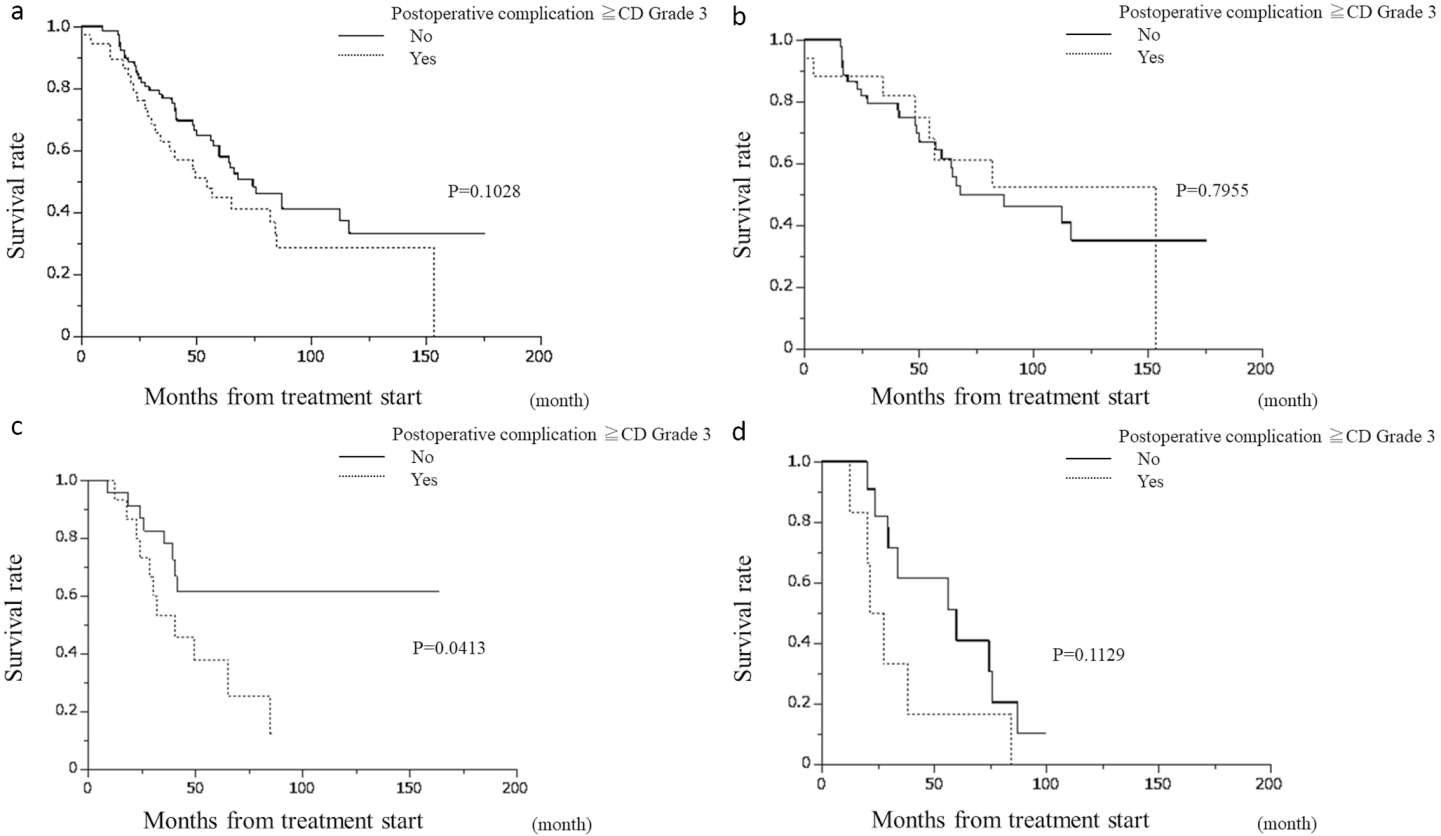
Survival curves compared by occurrence of postoperative complications. There was no significant difference overall (a) or in up-front surgery group (b). The prognosis of patients with complications was significantly worse in neoadjuvant chemotherapy group (c). In conversion surgery group (d), the prognosis tended to be worse in patients with complications; however, the difference was not statistically significant

Figure 1 depicts the overall survival after the start of treatment, showing that Group S had a significantly better prognosis than Group C, while Group C had a significantly better prognosis than Group I. The prognosis of Group N was not significantly different from that of Group C. Figure 2 illustrates the relapse-free survival after resection in Groups S, N, and C where R0/R1 resection was performed, indicating that Group C had a significantly shorter RFS than Groups S and N. In Group C, 15 patients experienced recurrence; the exceptions were one patient who died of liver failure after surgery and one who died of other diseases. No patients achieved a cure. A comparison between the occurrence of postoperative complications and the prognosis for CD grade ≧ 3 is shown in Fig. 3. There was no significant difference overall or in Group S. The prognosis of patients with complications was significantly worse in Group N. In Group C, the prognosis tended to be worse in patients with complications; however, the difference was not statistically significant.

When Group C was compared to Group I in patients initially deemed unresectable (Table 4), a univariate analysis showed that Group C had included significantly more male patients, patients with left-sided primary cancer, patients with single organ metastasis, patients with no peritoneal dissemination, and patients whose CA19-9 levels were within the normal range. In a multivariate analysis of these factors, a CA19-9 level within the normal range was independently associated with conversion surgery.

**Table 4:** Clinicopathological factors associate with conversion surgery

	Group C^†^	Group I^‡^	Univariate analysis	Multivariate analysis	
			*P* value	*P* value	Odd Ratio
Gender			0.0467	0.0580	
Male	13 (76)	113 (51)			3.62(0.96–13.7)
Female	4 (24)	109 (49)			Ref
Age (median)	63 (43–78)	67 (26–95)	0.1123		
Side of primary tumor			0.0072	0.0742	
Righ side	1 (6)	84 (38)			Ref
Left side	16 (94)	138 (62)			6.64(0.83-53.0)
Histological type			0.2074		
Well or moderately differenciated adenocarcinoma	16 (94)	173 (79)			
Others	1 (6)	47 (21)			
Clinical T			0.4053		
T1/T2/T3	6 (35)	57 (26)			
T4a/T4b	11 (65)	164 (74)			
Clinical N			1.0		
N-	3 (18)	35 (17)			
N+	14 (82)	170 (83)			
Metastatic organs			0.0391	0.3366	
1 or 2	10 (59)	75 (34)			1.71(0.57–5.12)
More than 3	7 (41)	96 (43)			Ref
Hepatic metastasis			0.5924		
Yes	13 (76)	150 (68)			
No	4 (24)	72 (32)			
Pulmonary metastasis			0.0703		
Yes	3 (18)	95 (43)			
No	14 (82)	127 (57)			
Peritoneal metastasis			0.0363	0.1328	
Yes	2 (12)	83 (37)			Ref
No	15 (88)	129 (63)			3.31(0.69–14.8)
Distant lymph node metastasis			0.8291		
Yes	6 (35)	72 (33)			
No	11 (65)	148 (67)			
CEA			0.0726		
Normal	6 (38)	38 (18)			
Elevated	10 (62)	177 (82)			
CA19-9			0.0164	0.0398	
Normal	10 (63)	71 (33)			3.12(1.03–9.38)
Elevated	6 (37)	145 (67)			Ref
RAS status			0.4899		
Wild	10 (67)	107 (57)			
Mutant	5 (33)	79 (42)			
Not evaluated	0 (0)	1 (1)			
BRAF status			0.6136		
Wild	13 (100)	241 (91)			
Mutant	0 (0)	22 (8)			
Not evaluated	0 (0)	1 (1)			

In Group C and Group I column, data are presented as n (%) or median. In the odd ratio column, data are presented as Odd Ration (95%CI).

^†^Group C: Patients who underwent conversion surgery. ^‡^Group I: patients who received chemotherapy only

## Discussion

Conversion rates in large clinical trials for unresectable advanced recurrent colorectal cancer have been found to be remarkably high. Several reports suggest that > 50% of colorectal cancers may be resectable, which may present a risk of disseminating misleading information to the public by implying that more than half of such colorectal cancers could be surgically treated even if initially deemed unresectable. The review by Bolhuis et al. [1]. focused exclusively on liver metastases in colorectal cancer and delineated the resectability criteria for such metastases. While the expected residual liver volume after resection is a criterion included in most reports, other criteria vary from trial to trial. In decision-making in relation to resectability, most reports involved a multidisciplinary team, but some reports indicated that the decision was made at a meeting of the medical team (as we made our decisions), while others did not describe the method of decision making. Given the inconsistencies in determining resectability and the criteria used, cases deemed unresectable in one trial may be judged as resectable in another [9]. We deem a tumor to be resectable if its resection is technically feasible and preservation of the vital organ function after resection can be ensured. Marginally resectable cases, in which the tumor on the dissected surface may be exposed but is not unresectable, are considered resectable. Preoperative chemotherapy for such cases is treated as neoadjuvant chemotherapy and when the tumor is resected it is not considered to be conversion surgery. Some reports treat cases that are technically resectable but oncologically unsuitable for resection as unresectable [10], so caution must be exercised when interpreting the conversion rate. If only cases that are technically unresectable and resected after chemotherapy are considered conversion surgeries, the actual conversion rate may be similar to the value we reported here.

Postoperative recurrence was frequently observed in patients who underwent conversion surgery in our study, and the RFS was significantly shorter than in patients who underwent upfront surgery or who underwent resection after neoadjuvant chemotherapy. Nonetheless, OS was better than in patients who received chemotherapy alone. Similar to the present investigation, Adam et al. reported that conversion surgery (termed “rescue surgery” in their report) exhibited a poorer prognosis in comparison to patients who received initial resection, but exhibited a more favorable outcome in comparison to unresectable cases [11]. If unresectable cases become resectable after chemotherapy, they may have a better prognosis in comparison to unresectable cases managed without surgery. However, surgery may be curative in such cases, and good outcomes that are difficult to achieve with chemotherapy alone have been reported [11, 12]. Based on a reanalysis of the cases in the Fire-3 trial, Modest et al. reported that the prognosis of patients who underwent surgery after being deemed resectable was better than that of patients who were deemed resectable but who did not undergo surgery [9]. Unfortunately, we have not yet experienced any curatively treated cases. However, considering the improvement in the prognosis, conversion surgery is still worthwhile, even though recurrence is frequently observed.

Conversion surgery is more likely to be invasive in patients with multiple metastases or invasion of adjacent organs due to the original unresectability of the disease. While there are reports on adverse events associated with chemotherapy administered before surgery, there are few reports on the safety of conversion surgery itself. Fukuoka et al. reported that liver resection after chemotherapy may be conducted safely without increased risk if the usual criteria are followed [13], thereby indicating that there is no requirement to exercise restraint in undertaking the procedure solely on the basis that it is performed after chemotherapy. Similarly, in the present study, chemotherapy had no effect on surgical safety. However, when the prognosis of patients with CD grade ≥ 3 complications was compared to the prognoses of the other groups, the prognosis of Group N was significantly worse, and the same trend was observed in Group C, although there was no significant difference in Group S (Fig. 3). The occurrence of postoperative complications has been reported to have a negative impact on the prognosis [14–16]; however, in this study, such an impact was only observed in post-chemotherapy cases. No reports have specifically addressed the prognostic impact of postoperative complications after chemotherapy, and future studies are warranted.

The multivariate analysis revealed that left-sided primary cancer and normal CA19-9 levels were the factors associated with the achievement of conversion surgery. Nozawa et al. reported single organ metastasis, liver metastasis cases, and use of molecular targeted therapy as independent factors [17], which differs from the results of our study. We extracted factors that are useful for prediction at the stage of treatment initiation without the addition of treatment factors, which may have contributed to the different results. Most other reports were limited to liver metastases, which makes it difficult to compare the results of the present study with those of other studies involving a wide range of unresectable colorectal cancer.

The present study was associated with several limitations, including the fact that it is a retrospective study of a small number of patients at a single institution. The small number of cases, especially in Group C, may have prevented a thorough statistical analysis. We believe that it is necessary to increase the number of cases and re-examine this issue in the future. While the liver is the most common site of metastasis in colorectal cancer, cases of liver metastasis alone are limited, and there are many cases of extrahepatic metastasis. The present study is significant because there have been few reports that included extrahepatic metastasis.

In conclusion, the present study reported that the conversion rate was 7.1% in patients with advanced colorectal cancer with distant metastasis, including unresectable nonhepatic metastasis, when technically unresectable was the criterion for unresectability. Notably, the prognosis was more favorable than that of unresectable cases treated solely with chemotherapy. Of note, among unresectable cases, patients with left-sided primary cancer and normal CA19-9 levels appear to be suitable candidates for conversion surgery.

### Electronic supplementary material

Below is the link to the electronic supplementary material.

Supplementary Material 1

Supplementary Material 2

## Data Availability

Data is provided within the manuscript or supplementary information files.
